# A Scoping Review of Pediatric Healthcare Built Environment Experiences and Preferences Among Children With Disabilities and Their Families

**DOI:** 10.1177/19375867231218035

**Published:** 2023-12-21

**Authors:** Clarissa Yu, Emi Wong, Juliana Gignac, Meaghan Walker, Timothy Ross

**Affiliations:** 1 Bloorview Research Institute, Holland Bloorview Kids Rehabilitation Hospital, Toronto, Ontario, Canada; 2 Department of Geography and Planning, University of Toronto, Ontario, Canada; 3 Rehabilitation Sciences Institute, University of Toronto, Ontario, Canada

**Keywords:** child, disability, hospital, design, built environment

## Abstract

**Background:**

Some children with disabilities (CWD) frequently visit pediatric healthcare settings for appointments. Their age, disability, and regular visits mean that they have unique experiences in healthcare settings. While previous research has explored the clinical experiences of CWD, little is known about their experiences of pediatric healthcare built environments, even though these environments may play an important role in shaping their perceptions of care. Given the significant time that some CWD and chronic illnesses (e.g., cancer) spend in healthcare environments and the impact that these settings can have on their experiences, the knowledge gap concerning how they view, and experience healthcare built environments demands attention.

**Objective:**

To explore how CWD and their families experience pediatric healthcare built environments.

**Methods:**

A scoping review was conducted by searching five health science and interdisciplinary literature databases using terms related to children, disability, healthcare, and built environment. The search identified 5,397 records that were screened independently by three reviewers.

**Results:**

Nineteen studies met inclusion criteria and were considered in the final review. Findings indicate that CWD and their families value healthcare built environment features that support social engagement, patient privacy, and parental presence. Further, the age of a child was highlighted as an important factor that influences the built environment preferences of CWD.

**Conclusions:**

CWD and their families prefer certain healthcare built environment features to optimize their experiences in these settings. Healthcare designers can leverage these findings to advance more inclusive pediatric healthcare spaces to improve care and the quality of life for CWD and their families.

## Introduction

Pediatric healthcare built environments have a significant influence on how children experience care (Jiang, 2020). Evidence suggests that well-designed healthcare environments can enhance children’s experiences and mitigate stress that some associate with healthcare (Jiang, 2020). Architects and designers have begun to leverage empirical findings to inform the design of therapeutic healthcare spaces with a view to optimize the quality of patient care. This process is referred to as evidence-based design (EBD; Ulrich et al., 2008). Considering patient experiences is a critical component of EBD, as it helps to ensure that spaces are designed and optimized to meet the needs and preferences of their intended users (Browall et al., 2013; MacAllister et al., 2016).

To help inform EBD processes, past literature reviews (Gaminiesfahani et al., 2020; Pauli Bock et al., 2021) have sought to understand how populations experience pediatric healthcare built environments. Pauli Bock et al. (2021) conducted a review focused on understanding how pediatric hospital occupants experience their built environments; they found that different design elements have implications for reported stress and anxiety among children, parents, and staff. For example, design features supporting natural lighting, space for visitors, and noise reduction were associated with greater built environment satisfaction (Pauli Bock et al., 2021). Another review by Gaminiesfahani et al. (2020) identified nine built environment themes that contribute to the creation of therapeutic healthcare spaces for children and their families. These themes include considerations related to colors and decor, access to the outdoors, lighting, noise, and play, which were identified as important built environment components that promote therapeutic healthcare spaces (Gaminiesfahani et al., 2020). While these reviews have contributed significantly to the advancement of EBD processes in pediatric healthcare design, most studies to date have examined the built environment experiences among the general pediatric population, without considering the diverse experiences among specific subgroups of children.

Children with disabilities (CWD) represent one population that may have unique experiences within healthcare built environments. Evidence suggests that CWD, including those with chronic illnesses, visit healthcare spaces more frequently than their same-aged peers without disabilities (Arim et al., 2017; Kim et al., 2020). One estimate found that the emergency department (ED) and inpatient services in the United States are used approximately 1.8 times more often by children with developmental disabilities than other children (Lindgren et al., 2021). Given that some CWD spend considerably more time in healthcare spaces, it is alarming that remarkably little research has explored their experiences within pediatric healthcare built environments. To further advance EBD processes that support the creation of accessible and inclusive healthcare spaces, we conducted a scoping review to explore how CWD and their families experience and view pediatric healthcare built environments. This scoping review specifically engages the research question, “what is known about how CWD and their families experience pediatric healthcare built environments?”

***To further advance EBD processes that support the creation of accessible and inclusive healthcare spaces, we conducted a scoping review to explore how CWD and their families experience and view pediatric healthcare built environments***.

## Method

Our review process was guided by a scoping review methodology as outlined by Arksey and O’Malley (2005). From June 1 to 13, 2022, we searched five databases for peer-reviewed articles covering a 32-year period from 1990 to 2022. We selected 1990 as the start date for our review period to align with the 1990 *Americans with Disabilities Act* (ADA). The ADA prompted widespread consideration of built environment experiences of individuals with disabilities and thus serves as a suitable start date for this review. We applied our searches to five databases that we viewed as having content relevant to our research question: CINAHL via EBSCO, Medline via OVID, PsycInfo via OVID, Scopus, and the Web of Science Core Collection. These five databases cover medical, psychology, nursing, social sciences, and other disciplines, which helps to ensure a comprehensive search of interdisciplinary literature that may offer relevant knowledge and viewpoints on pediatric healthcare built environments.

### Search Strategy

Guided by our research question, we constructed a comprehensive search strategy. The Boolean operator “AND” was used to combine the following four topics that we viewed as being central to our research question: (1) children, (2) healthcare, (3) built environment, and (4) disability. We applied an “OR” Boolean operator between the terms for each topic so that our search would capture synonymous terms associated with each topic. Truncation searches (e.g., via the application of asterisks) were used to expand the search to include words with varying suffixes related to a root word. After careful revision in consultation with a qualified health sciences librarian, the following search function was created:

1. (“child*” OR “teen*” OR “adolescen*” OR “youth” OR “kid” OR “kids” OR “young people” OR “young person*” OR “pediatric*” OR “paediatric*” OR “juvenile” OR “son” OR “sons” OR “daughter*” OR “girl” OR “girls” OR “boy” OR “boys”)
2. AND (“healthcare” OR “health care” OR “medicine” OR “rehab*” OR “medical treatment*” OR “patient care” OR “primary care*” OR “inpatient care” OR “outpatient care” OR “urgent care” OR “ambulatory care” OR “palliative care” OR “occupational therap*” OR “physical therap*” OR “physiotherap*” OR “speech-language patholog*”)
3. AND (“built environment*” OR “architectur*” OR “interior design*” OR “exterior design*” OR “spatial design*” OR “public space*” OR “waiting room*” OR “hospital room*” OR “patient* room*” OR “emergency room*” OR “playroom*” OR “play room*” OR “lounge*” OR “doctor’s office*” OR “snoezelen” OR “clinic* environment*” OR “clinic design*” OR “clinic* space*” OR “hospital environment*” OR “hospital design*” OR “hospital space*” OR “placemaking” OR “place-making” OR “placekeeping” OR “place-keeping” OR “hospital garden*” OR “healing garden*” OR “therapeutic garden*” OR “sensory garden*”)
4. AND (“disab*” OR “impair*” OR “handicap*” OR “special need*” OR “disorder*” OR “chronic condition*” OR “chronic illness*” OR “medical complexit*” OR “syndrome” OR “spina bifida” OR “cerebral palsy” OR “muscular atrophy” OR “muscular dystrophy” OR “autis*” OR “asperger*” OR “seizure*” OR “epilepsy” OR “amput*” OR “spinal injur*” OR “brain injur*” OR “cancer” OR “cystic fibrosis”)

The above search function was applied to titles, abstracts, and key words within Scopus and Web of Science databases. With the support of a health sciences librarian, this baseline string was modified for Medline, CINAHL, and PsycInfo. As these databases use subject headings to index literature, modifying the search function to incorporate subject headings helped to ensure the search was tailored for each database to maximize the number of relevant results.

### Eligibility Criteria

The records were screened with attention to five inclusion criteria:

1. published between 1990 and June 2022,
2. written in English,
3. peer-reviewed,
4. focused on children/youth with disabilities (including chronic illnesses and cancer) aged 5–18 years (inclusive) and/or their families, and
5. focused on experiences with the built environment in primary healthcare settings.

While the pediatric population age range is 0–18 years, we opted to focus this review on CWD aged 5–18 years. Given their young age and development stages, infants and children aged 0–5 years (and their families) have notably different healthcare needs and different healthcare environment experiences and preferences. We focused on CWD aged 5–18 years to allow for a more comprehensive and in-depth exploration of this age group’s experiences and preferences regarding pediatric healthcare spaces. Exploration of the healthcare environment experiences and preferences of CWD aged 0–5 years and their families is warranted in future research.

Our application of the 5–18 years age range criterion was applied flexibly. That is, some studies had populations with age periods that extended beyond our age range; however, if roughly half or more of a study’s participants were within the 5–18 years age range, the study was included to avoid missing relevant built environment experiences. Further, our consideration of disability extended to chronic illnesses, such as cancer, as these children may spend substantial time in healthcare environments and their perspectives are important to consider (de Flon et al., 2021).

The following criteria were used to exclude irrelevant studies:

1. focused on experiences of healthcare providers or adults unrelated to CWD,
2. focused on experiences in secondary healthcare settings (dental or psychotherapy), and
3. conference proceedings, dissertations, and commentaries/position statements.

### Screening

**Figure 1.**
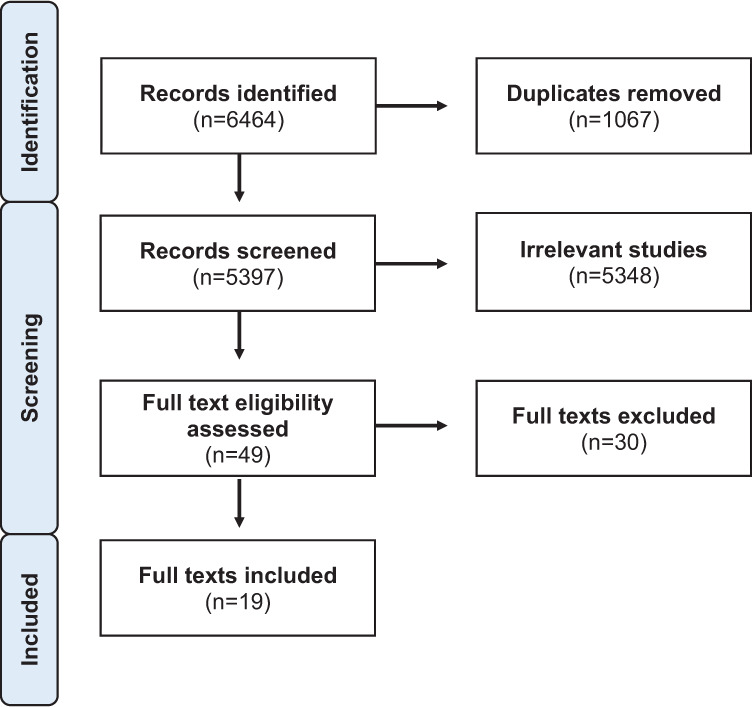
Preferred Reporting Items for Systematic Reviews and Meta-Analyses (PRISMA) diagram of the record screening and identification process.

By running our search across the five databases, we identified a total of 6,464 records (see Figure 1). Records were uploaded to the Covidence website, which offers software for screening records for literature reviews. Through this process, 1,067 duplicate records were removed. Three reviewers (C.Y., E.W., and J.G.) independently screened the titles and abstracts of the remaining 5,397 records. Records required a consensus between two independent reviewers to be removed as irrelevant or to be advanced to full-text screening, which is in line with scoping review best practices (Arksey & O’Malley, 2005). This process yielded 5,348 irrelevant records for removal, and 49 records for full-text screening. Of the 49 full texts assessed for eligibility, 30 texts were excluded. Texts were excluded for reasons such as focusing on the wrong population or not reporting on built environment experiences. Texts were also excluded if the full text was unavailable for review. The remaining 19 texts were included.

There were 91 and 14 screening conflicts at the title/abstract and full-text screening stages, respectively. Conflicts were resolved via discussions between the two conflicting reviewers (C.Y., E.W., or J.G.). A third reviewer (C.Y., E.W., J.G., or T.R.) was consulted if the two reviewers were unable to agree. Additionally, during the screening process reviewers identified nine literature reviews that had some peripheral relevance to our research question. Reference sections of the review papers were mined for additional articles but none were identified for inclusion in this review.

## Results

A total of 19 studies met inclusion criteria. Table 1 presents a description of each study’s population, location, aim, methods, and relevant built environment findings. Studies were conducted in the United States (*n* = 5), Canada (*n* = 3), the United Kingdom (*n* = 2), the Netherlands (*n* = 1), Iran (*n* = 1), Ireland (*n* = 1), Italy (*n* = 1), Norway (*n* = 1), Turkey (*n* = 1), Saudi Arabia (*n* = 1), Spain (*n* = 1), and Israel (*n* = 1). The study by Rollins (2009) collected data from two hospitals, located in the United States (US) and United Kingdom (UK) and was included in both categories for the US and UK above. The location of the study by Peeters et al. (2018) was not specified (*n* = 1). Twelve studies employed qualitative methods, including observations, interviews, and arts-based activities (*n* = 12). Four studies used quantitative methods, such as the visual analogue scale, quantitative surveys, and multivariate modeling (*n* = 4). Three studies used a mixed methods approach involving a combination of qualitative and quantitative methods (*n* = 3).

**Table 1.** Summary of Papers Included for Review.

**Study**	**Population and Location**	**Description**	**Built Environment Findings**
(1) Biddiss et al. (2018)	*Population:* 310 children (and their parents) with disabilities (e.g., cerebral palsy, autism spectrum disorders [ASD], spina bifida), 5–19 years old *Location:* Waiting space in a pediatric hospital’s outpatient clinic, Canada	*Aim:* To evaluate the impact of interactive media on children and families’ experiences in a pediatric waiting room *Methods:* Randomized controlled trial, questionnaires, and observations	Waiting spaces with media are perceived to be more fun by children and parents. Media also increases parental satisfaction with the space. Children report lower anxiety in waiting spaces when interactive media is present.
(2) Biddiss et al. (2019)	*Population:* 271 children with disabilities (e.g., cerebral palsy, ASD, spina bifida), 5–19 years old *Location:* Waiting space in a pediatric hospital’s outpatient clinic, Canada	*Aim:* To determine the use and value of personal versus hospital-provided distractions in waiting spaces *Methods:* Randomized controlled trial, questionnaires, and observations	Children interacted with hospital-provided distractions (e.g., aquarium, interactive media) more than personal ones (e.g., phones). Age influences children’s activity choice in waiting spaces. Children’s anxiety levels differ according to their activity choice.
(3) Boyd & Hunsberger (1998)	*Population:* Six children with chronic illnesses, 10–13 years old *Location:* Inpatient units of a pediatric hospital, Canada	*Aim:* To identify and describe frequently hospitalized children’s sources of stress in hospital, coping strategies, and support from others that enables coping *Methods:* Drawings, journals, and semi-structured interviews	Lack of privacy and noise can be stressors. Built environment features supporting privacy, play, parental presence, and access to the outdoors such as private rooms, playrooms, and windows can enable coping.
(4) Corsano et al. (2015)	*Population:* 50 children (and 48 of their adult family members) with cancers, tumors, and other diseases, 7–15 years old *Location:* Waiting room in a pediatric oncology and hematology clinic, Italy	*Aim:* To describe the experiences of children and families in a hospital waiting room *Methods:* Semi-structured interviews, drawings, **and** questionnaires	Children often experience boredom while waiting but not anxiety and other negative emotions. Adults also experience boredom but report greater anxiety and concern for their children. Ninety percent of adult family members recommend including positive distractions in waiting rooms.
(5) Ebrahimpour et al. (2021)	*Population*: 20 children with cancer, 6–12 years old *Location:* Oncology ward in a pediatric hospital, Iran	*Aim:* To identify and describe hospitalized children’s sources of hope in an oncology ward *Methods:* Photovoice, semi-structured interviews	Playrooms offer hope by serving as places to socialize and distract from disease and boredom. Nature elements (e.g., plants, nature paintings) and access to the outdoors provide hope. Hospital interiors can cause children to feel trapped.
(6) Ekra & Gjengedal (2012)	*Population:* Nine children with type 1 diabetes, 7–12 years old *Location:* Pediatric ward and outpatient clinic in a hospital, Norway	*Aim:* To describe how recently diagnosed children experience hospitalization and how the hospital environment affects their experiences *Methods:* Observations, interviews, and photovoice	Child-oriented decor, privacy afforded by single rooms, parental presence, and visits from others provide a sense of comfort to children in what can feel like an alien environment. Illness can impact how children perceive the environment.
(7) Gürcan & Atay Turan (2021)	*Population:* 12 children with cancer, 10–17 years old *Location* **:** Pediatric oncology and hematology unit of hospital, Turkey	*Aim:* To identify and describe what hospitalized children expect from a therapeutic hospital environment *Methods:* Semi-structured interviews	Children value hospital features that accommodate privacy (e.g., single rooms), age (e.g., appropriate colors and decor), and play (e.g., designated playrooms). Children prefer environments conducive to sleep and outdoor access.
(8) Hamdan et al. (2016)	*Population:* Children with cancer and their families *Location:* Pediatric unit of a hospital’s cancer center, Saudi Arabia	*Aim:* To determine the effectiveness of environmental modifications to improve patient and their families’ satisfaction with the hospital environment *Methods:* Surveys, modifications to environment	Play and waiting room spaces were improved and nature and zoo-themed paintings were installed in hallways, waiting and patient rooms. Modifications improved patient and families’ satisfaction with the unit’s appearance, decor, play, and waiting areas.
(9) Lambert et al. (2014a)	*Population:* 55 children with chronic or acute illnesses, 5–8 years old *Location* **:** Three hospitals in Ireland	*Aim:* To describe children’s perceptions and preferences of social spaces in hospitals *Methods:* Semi-structured interviews with art activities	Children prefer a variety of play activities in designated spaces and incorporated throughout the hospital. Children value spaces that support socialization. Technology may play a role in advancing these needs.
(10) Linder & Seitz (2017)	*Population:* 50 children with cancer, 7–18 years old *Location*: Inpatient ward in a pediatric hospital, United States	*Aim:* To identify and describe what hospitalized children perceive as bothersome aspects of the hospital environment *Methods:* Written responses to two open-ended questions	Built environment features including, small room size, decor, temperature, and noise level are bothersome and can be improved. Children feel isolated in hospital and report a lack of activities to keep occupied.
(11) Miralles et al. (2016)	*Population:* Nine adolescents with cancer, 15–19 years old *Location:* Pediatric oncology wards, Spain	*Aim:* To identify what hospitalized adolescents need to engage in meaningful activities and to describe potential barriers limiting these activities. *Methods:* Observations, in-depth interviews	Lack of privacy in shared rooms prevents adolescents from undertaking some activities of daily living. Adolescents report a lack of age-appropriate recreational activities and ward space.
(12) Peeters et al. (2018)	*Population:* 10 adolescents with cancer, 14–25 years old *Location:* Multiple hospitals	*Aim:* To describe how adolescents experience hospitalization and how this experience is affected by the built environment. *Methods:* Semi-structured retrospective interviews	Adolescents value hospital environments that support privacy (e.g., single rooms), socialization with patients and visitors, outdoor access, and provide natural light and distractions. Home-like features improve comfort.
(13) Rollins (2009)	*Population:* Children with cancer and their families *Location:* Two pediatric hospitals, United Kingdom and United States	*Aim:* To determine how hospital design and policies affect children and their families’ ability to cope with hospitalization. *Methods:* Analysis of hospital floor plans and photos, relevant guidelines, and previously conducted interviews, observations, and children’s drawings	Shared spaces facilitating socialization are valued by children and families. Shared spaces located off the ward are used less. Privacy is important for coping but valued differently by children of different ages. Spaces can be adapted by staff to meet the needs of patients and families.
(14) Sachs & Nasser (2009)	*Population:* 19 parents of children with intellectual disabilities, 4–17 years old *Location:* Pediatric assisted living facility, Israel	*Aim:* To describe how parents experience and perceive family gatherings in the Snoezelen room of a residential facility *Methods:* Observations, in-depth semi-structured interviews	Most parents consider the Snoezelen room a relaxing and quiet escape from the facility. Other facility spaces are loud and lack privacy, preventing intimate family gatherings. Snoezelen rooms facilitate family bonding.
(15) Sherman et al. (2005)	*Population:* 1,400 people using the gardens at the cancer center *Location:* Three gardens at a pediatric hospital’s cancer center, United States	*Aim:* To observe and describe the usage of gardens at a pediatric hospital’s cancer center *Methods:* Observations, visual analogue scale-based questionnaire	Pediatric patients represent the smallest proportion of garden users despite child-centered garden designs. Children engaged with garden elements more than other users. Correlations between garden occupancy and window use suggest privacy concerns.
(16) Sherman-Bien et al. (2011)	*Population:* 90 children (and 149 of their parents) with hematology or oncology diagnoses, 8–17 years old *Location:* Hematology–oncology inpatient unit of a pediatric hospital, United States	*Aim:* To determine how objective features of healthcare built environments correlate to health outcomes and satisfaction with the received healthcare and the built environment *Methods:* Questionnaires, structural equation modeling	There exists a significant positive correlation between features of and satisfaction with the built environment. Additional correlations exist between satisfaction with the built environment and the following two variables for parents: health outcomes and satisfaction with healthcare.
(17) Vollmer & Koppen (2021)	*Population:* 22 parents of hospitalized pediatric oncology patients, 3–5+ years old *Location:* Inpatient rooms in two pediatric oncology wards, Netherlands	*Aim:* To determine how inpatient room design influences parental stress during a child’s hospitalization *Methods:* Observations, questionnaires, visual analog scale	Suboptimal patient room designs can contribute to parental stress during their child’s hospitalization. Room designs allowing privacy and healthy separation between parent and child may reduce parental stress.
(18) Wharton et al. (2005)	*Population:* 25 parents of children with developmental disabilities (e.g., cerebral palsy, ASD), 3–18 years old *Location:* National Health Service hospitals, United Kingdom	*Aim:* To survey parents’ perceptions about the accessibility of NHS hospitals and services for children with disabilities *Methods:* Questionnaire done via semi-structured interview	Parents of wheelchair users identified accessibility issues with parking lots, waiting rooms, and other spaces (e.g., hallways). Waiting rooms and procedures are not sensory-friendly. Parents prefer privacy and more accessible hospital wards.
(19) Wood et al. (2019)	*Population:* Children with ASD or sensory processing disorders (SPD) and their families *Location:* Pediatric emergency department (ED), United States	*Aim:* To design and implement modifications to a pediatric ED to improve the experiences of children with ASD/SPD and their families. *Methods:* Discussions and questionnaire	Busy waiting rooms can be stressful for children with ASD/SPD. Sensory-friendly changes to ED procedures and the addition of distractors can improve children and their families’ experiences.

Most studies included both children under 10 years old and adolescents aged 10 years and above (*n* = 9). Of the studies including only children under or over age 10, four studies focused on adolescents (*n* = 4), while only two studies focused on children under 10 years old (*n* = 2). Children’s ages in four studies were not reported (*n* = 4). Although our inclusion criterion for age range was 5–18 years, the study by Peeters et al. (2018) included young adults (up to age 25), and the study by Vollmer and Koppen (2021) included children under Age 5. These studies were included because a significant proportion of their populations included children within the 5–18 years age range criterion. The types of disability represented include pediatric cancer and/or hematological disease (n = 11), developmental disabilities (n = 5), unspecified chronic illnesses (n = 2), and type 1 diabetes (n = 1). While nine studies focused on the experiences of children in healthcare spaces (*n* = 9), seven studies included the experiences of both children and family members (*n* = 7). Three studies focused exclusively on the experiences of parents (*n* = 3).

Almost all studies considered the experiences of hospital environments (*n* = 18), where nine focused on inpatient (*n* = 9), four focused on outpatient (*n* = 4), and five focused on both inpatient and outpatient experiences (*n* = 5). One study (Sachs & Nasser, 2009) examined experiences within a pediatric assisted living facility (*n* = 1). Within these spaces, experiences of waiting rooms, playrooms, and inpatient rooms were most reported in the literature. One study (Sherman et al., 2005) reported on the experiences of children and their families in outdoor gardens at a pediatric cancer treatment center.

## Discussion

Through our review of the 19 included studies, we identified three key themes that reflect the viewpoints of CWD and their families on the topic of pediatric healthcare built environments. These themes are (1) The Importance of Social Opportunities, (2) Enhancing Healthcare Experiences via Architectural and Interior Design, and (3) Missing Voices: Young Children and Developmental Disability.

### The Importance of Social Opportunities

Social opportunities that enable both children and parents to engage with others represent an important component of pediatric healthcare spaces (McLaughlan, 2018). Social engagement has been found to help children and their families cope with illness and hospitalization, and many have reported that sharing experiences with others facing similar challenges offers therapeutic benefits (Kelada et al., 2021; McLaughlan, 2018). The included studies are consistent with these findings, as both CWD and their parents valued the opportunity to share their experiences with peers facing similar health-related circumstances (Ebrahimpour et al., 2021; Gürcan & Atay Turan, 2021; Rollins, 2009). However, the built environment plays an important role in shaping social engagement, as its spaces can facilitate or hinder social opportunities to engage with others (Devlin et al., 2016).

The reviewed studies suggest that social opportunities for CWD within pediatric healthcare settings are mostly considered and offered in the context of designated play/recreation rooms. Children value these spaces as opportunities to form friendships, distract from hospitalization, and to have fun (Boyd & Hunsberger, 1998; Ebrahimpour et al., 2021; Gürcan & Atay Turan, 2021; Lambert et al., 2014a). However, the focus on designated play/recreation rooms suggests that social opportunities beyond these spaces have received little consideration in the design and operation of pediatric healthcare settings. This is concerning given evidence in the literature that indicates children are interested in having opportunities to play and socialize incorporated throughout their healthcare environments, such as at their bedside or in hallways (Lambert et al., 2014a, 2014b; Rollins, 2009).

Having play and social opportunities beyond the boundaries of designated play spaces may be particularly important for those who cannot readily access these play spaces, such as those whose conditions require them to remain in their hospital room or within the nearby vicinity (Lambert et al., 2014b). Child-life specialists currently play an important role in facilitating bedside play to fulfill this unmet need when designated spaces are inaccessible to children (Burns-Nader & Hernandez-Reif, 2016). However, seeking to expand social opportunities beyond designated spaces, for example, modifying the bedside environment to better support children and child-life specialists during play, could help to enhance access to critical play and social opportunities for CWD and their families who regularly visit and spend more time in healthcare settings (Arim et al., 2017; Kim et al., 2020).

***...seeking to expand social opportunities beyond designated spaces, for example, modifying the bedside environment to better support children and child-life specialists during play, could help to enhance access to critical play and social opportunities for CWD and their families who regularly visit and spend more time in healthcare settings***.

Considering social spaces beyond designated play/recreational rooms may also help to create spaces that support socialization during visiting hours. Scholars (Ekra & Gjengedal, 2012; Gürcan & Atay Turan, 2021; Peeters et al., 2018) have reported that children desire connections with relatives and peers while in hospital. Such connections can offer them comfort and have been found to reduce feelings of isolation (Ekra & Gjengedal, 2012; Gürcan & Atay Turan, 2021). However, little scholarly attention has been given to spatial designs that support socializing during visits in pediatric healthcare spaces. In one study, adolescents felt discouraged from inviting peers to visit them citing a lack of appropriate hospital space (Peeters et al., 2018). To ensure that CWD and their families have adequate space to socialize with visitors, future research should consider design opportunities for children and youth to socialize beyond the boundaries of play/recreation rooms. Studies could consider how cafeterias, lounges, atria, gardens, and other common spaces found within pediatric healthcare environments can be leveraged to support opportunities for social engagement with peers and family.

### Enhancing Healthcare Experiences via Architectural and Interior Design

Architectural (e.g., floor plan layout, windows) and interior design (e.g., colors, decor, furniture) elements can help to mitigate stress and promote healing to enhance children’s experiences within healthcare spaces (Lambert et al., 2014b; Nasab et al., 2020). In fact, evidence suggests that specific healthcare design features have tangible implications on health outcomes, such as anxiety, which further emphasizes the importance of thoughtfully designed built environments (Sherman-Bien et al., 2011). With respect to interior design, previous literature has found that children value the use of blue and green colors, decorative paintings, and nature elements that create comfortable and home-like environments (Lambert et al., 2014b; Nasab et al., 2020). These findings are consistent with those of the literature we reviewed, which indicate that CWD appreciate the addition of colors, child-friendly decorations, and nature-themed elements in healthcare settings (Ebrahimpour et al., 2021; Ekra & Gjengedal, 2012; Gürcan & Atay Turan, 2021; Hamdan et al., 2016; Peeters et al., 2018). These interior design elements can help to transform hospitals from feeling cold and institutional into home-like environments that offer greater comfort and support (Ekra & Gjengedal, 2012; Peeters et al., 2018).

Architectural features can also enhance children’s healthcare experiences. Some of the included studies found that CWD value designs that support parental presence during a hospitalization by offering parents adequate space to rest and spend time with their children in patient rooms, as well as spaces designated for them to take breaks (Boyd & Hunsberger, 1998; Ekra & Gjengedal, 2012; Peeters et al., 2018). These results are consistent with previous studies, where children—with and without disabilities—identified parental presence as important to their healthcare experiences (Lambert et al., 2014b; Nasab et al., 2020). Despite the importance of parental presence, little research has questioned how healthcare centers’ architectural designs can accommodate and support them. One study found that the patient rooms in a children’s cancer center were designed suboptimally with little space for parents to relax and sleep, such that they contributed to parental stress during hospitalization (Vollmer & Koppen, 2021). Further, in a study by Wharton et al. (2005), parents reported a lack of appropriate space within inpatient wards to carry out self-care activities, such as eating or showering. This concern was echoed by Rollins (2009), who analyzed the social and privacy features of two hospitals and reported that there was inadequate space for parents to rest and have private conversations, beyond hallways. Recognizing the importance of parental presence to CWD and chronic conditions during hospitalizations, the architectural designs of pediatric healthcare spaces could be improved to account for and support parents. Providing parents with ample opportunities to provide input on their needs and preferences relating to visiting and staying at hospitals throughout design and planning processes could meaningfully contribute to creating pediatric healthcare spaces that better support parents. In turn, this could help parents to better support their children throughout their healthcare experiences.

In addition to architectural designs supporting the presence and comfort of parents, we found notable discussion in the literature around privacy-related architectural features. Many studies reported that CWD undergoing inpatient care preferred single rooms (Boyd & Hunsberger, 1998; Ekra & Gjengedal, 2012; Gürcan & Atay Turan, 2021; Peeters et al., 2018; Rollins, 2009). These rooms offer privacy which can cultivate a sense of comfort in an unfamiliar hospital environment and in turn help them to cope with hospitalization (Boyd & Hunsberger, 1998; Ekra & Gjengedal, 2012; Peeters et al., 2018; Rollins, 2009). This common finding clearly suggests that children value privacy in healthcare settings. In fact, it is well documented that privacy is an important consideration in healthcare spaces, as it can improve patient satisfaction and provide patients with a sense of control over their environment (Alalouch et al., 2016; Lambert et al., 2014b). In the reviewed literature, privacy is mostly discussed in the context of single-patient rooms. However, there is little discussion about specific design features within these rooms that can enhance the privacy, such as room configurations, soundproofing, lines of sight into/and out of rooms, and window shades. Further, little attention has been given to building design features beyond hospital rooms that may create opportunities for or feelings of privacy in healthcare spaces. Creating designated quiet or bookable spaces may function to support the privacy needs of children if their preference for single rooms cannot be satisfied. This was found by Sachs and Naser (2009), who reported that parents valued having access to quiet and private spaces, such as Snoezelen rooms, as these spaces provided invaluable family bonding opportunities otherwise unavailable within the healthcare facility. Exploring further ways in which privacy-related elements can be integrated into healthcare spaces—both within and beyond hospital rooms—could be beneficial to CWD and their wellbeing and to family relations.

Although children had similar preferences for architectural design features (e.g., all children valued parental presence and privacy in healthcare settings), the literature highlights differences between younger and older children. That is, age has consistently been found to influence children’s built environment preferences as they relate to parental presence and private spaces. Younger children preferred consistent parental presence, including overnight stays, whereas older adolescents did not always share this preference and valued their independence (Peeters et al., 2018). Regarding privacy, one study found that while adolescents indicated a preference for single rooms, younger children often gravitated toward the doorway of their room, which has been interpreted as attempts to include themselves in ward activities (Rollins, 2009). Other studies have similarly found that while both younger children and adolescents value privacy, this preference is stronger among adolescents (Ekra & Gjengedal, 2012; Miralles et al., 2016). In fact, a study by Miralles et al. (2016) found that the lack of privacy associated with shared rooms can discourage adolescents from engaging in some daily living activities. These findings suggest that privacy within healthcare spaces is important to both younger children and adolescents but more so to adolescents. Further, these differences between adolescents and children carry over to interior design preferences. Some adolescents disliked child-friendly decorations, such as drawings or paintings of animals, because they viewed them as childish (Peeters et al., 2018). Some children thought that the color and decor of a hospital room should reflect a patient’s age, with rooms for younger children having brighter colors and decor (e.g., rainbows, animals) whereas rooms for older children could have more mature color palates and themes (Gürcan & Atay Turan, 2021). Given the significant influence of age on children’s healthcare experiences, it would be beneficial for scholars and healthcare designers to consider how age-related preferences for parental presence, privacy, and room colors and decor could be incorporated into designs to ensure that healthcare environments are inclusive to all children. Future inquiry into interchangeable interior decor and adjustable room configurations to suit parental presence and/or privacy preferences could help to account for and accommodate individual preferences of children and adolescents living with disability.

***Given the significant influence of age on children’s healthcare experiences, it would be beneficial for scholars and healthcare designers to consider how age-related preferences for parental presence, privacy, and room colors and decor could be incorporated into designs to ensure that healthcare environments are inclusive to all children***.

### Missing Voices: Young Children and Developmental Disability

While children of different ages have unique experiences and preferences relating to healthcare built environments, relatively little attention has been given to younger children’s viewpoints on this topic. Of the 15 of 19 reviewed studies specifying children’s ages, only two studies focused primarily on the perspectives of children under 10 years of age. Nine studies considered the combined perspectives of both adolescents over Age 10 and younger children, but employed methods (e.g., questionnaires, qualitative interviews, written-answer questions) that suggest they were more so focused on understanding the perspectives of adolescents, as they were not particularly suitable for younger children. We say this because there is evidence indicating that conducting traditional question and answer interviews with young children, and obtaining quality answers from this group, typically requires specialized training and study designs (Fleer & Li, 2016; Ponizovsky-Bergelson et al., 2019). Having said this, three of the nine studies involved the use of arts-based methods to gather built environment experiences. To engage younger children about their built environment experiences and preferences, arts-based methods, such as photovoice (Ross, 2020; Ross et al., 2020; Wang & Burris, 1997) or draw-and-write activities (Backett-Milburn & McKie, 1999; Moola, 2020) are preferable, as they offer children a comfortable, alternative mode of communication for them to share their viewpoints (Ponizovsky-Bergelson et al., 2019; Tay-Lim & Lim, 2013). Beyond engaging younger audiences, arts-based activities can make study participation (or pediatric healthcare center design processes) more accessible to CWD. For example, arts-based activities can be invaluable in terms of providing children who communicate nonverbally an opportunity to share their experiences and preferences (Moola et al., 2022). Incorporating arts-based methodologies into future investigations may help to build a more comprehensive understanding of pediatric healthcare spaces for both younger children and those with certain disabilities. This may help to advance the architectural and interior design of pediatric spaces to better serve all children.

While all reviewed studies focused on the experiences of CWD, the experiences of children with cancer figured prominently in the literature (11 of the 19 total studies). Experiences of children with developmental disabilities were somewhat underrepresented (five of the 19 total studies). Those studies that did consider built environment experiences of children with developmental disabilities speak to the need for greater physical accessibility (Wharton et al., 2005) and to the importance of Snoezelen spaces (Sachs & Nasser, 2009). By interviewing parents of children with developmental disabilities, Wharton et al. (2005) found that children who use wheelchairs may face challenges with manual doors, inaccessible reception areas with desks that are too high, as well as fixed seating and small spaces in waiting rooms. Another study investigating the experiences of children with autism spectrum and sensory processing disorders (ASD/SPD) and their families reported that traditional waiting spaces in pediatric emergency departments can be stressful, whereas allowing children with ASD/SPD to wait in designated quiet spaces or private rooms can improve their experience (Wood et al., 2019). Biddiss et al. (2018, 2019) added to this waiting room design knowledge by reporting that integrating media into pediatric clinic waiting spaces can help to reduce anxiety for children with various developmental disabilities. These findings indicate that children with developmental disabilities and their families have unique experiences and challenges within healthcare built environments. However, it remains that little is known about this group’s experiences and preferences relating to key spaces (e.g., inpatient rooms) and how they would change their healthcare environments to better suit their needs and preferences. It would be practical for future studies to focus on understanding how children with developmental disabilities and their families experience pediatric healthcare environments to help inform the design of these spaces so that future spaces can better satisfy this group’s needs and preferences. Seeking knowledge from this group would be particularly beneficial, given that this group has unique spatial needs and preferences and may undergo frequent visits to healthcare spaces (Kim et al., 2020).

***It would be practical for future studies to focus on understanding how children with developmental disabilities and their families experience pediatric healthcare environments to help inform the design of these spaces so that future spaces can better satisfy this group’s needs and preferences***.

## Conclusion

This scoping review identified several built environment needs and preferences of CWD aged 5–18 years and their families. Integrating design features that promote opportunities for social engagement, create home-like environments, enable parental presence, and support patient privacy can enhance the ways in which children and their families experience pediatric healthcare spaces. Further, accounting for the ways in which children’s age impacts their built environment preferences may provide useful insights into how we can design healthcare spaces to be positively experienced by all children. Future research is needed to engage the knowledge gaps pertaining to the ways in which younger children and children with developmental disabilities experience their healthcare environments. These children have unique experiences of healthcare spaces and research should carefully engage and account for their perspectives to ensure that healthcare settings are inclusive of their needs. Pursuing these research directions may help healthcare designers to better understand, appreciate, and explore new methods through which they can engage CWD about their spatial needs and preferences. Greater engagement with CWD in research on the design of pediatric healthcare environments, and in the design processes, could help to advance more accessible and inclusive therapeutic healthcare spaces, improved quality of care, and optimal healing environments for CWD and their families.

***Greater engagement with CWD in research on the design of pediatric healthcare environments, and in the design processes, could help to advance more accessible and inclusive therapeutic healthcare spaces, improved quality of care, and optimal healing environments for CWD and their families***.

## Implications for Practice

- Specific architectural and interior design features within pediatric healthcare built environments can enhance the healthcare experiences of CWD and their families; designers can use knowledge of these features to create inclusive healthcare spaces and, in turn, improve care experiences.
- CWD and their families value spaces that support social engagement, parental presence, and patient privacy.
- The age of CWD is an important factor to consider during design processes because it affects their built environment preferences.
- Further research is needed to understand the healthcare built environment experiences of children with developmental disabilities, as their healthcare built environment experiences are underrepresented in the literature.
